# Limited Sampling Spatial Interpolation Evaluation for 3D Radio Environment Mapping

**DOI:** 10.3390/s23229110

**Published:** 2023-11-10

**Authors:** Antoni Ivanov, Krasimir Tonchev, Vladimir Poulkov, Agata Manolova, Atanas Vlahov

**Affiliations:** 1 Faculty of Telecommunications, Technical University of Sofia, 1000 Sofia, Bulgaria; k_tonchev@tu-sofia.bg (K.T.); vkp@tu-sofia.bg (V.P.); amanolova@tu-sofia.bg (A.M.); 2 Intelligent Communication Infrastructure Laboratory, Sofia Tech Park, 1784 Sofia, Bulgaria; ici-lab@sofiatech.bg

**Keywords:** interpolation, Kriging, radio environment maps, region of interest, volumetric measurements

## Abstract

The increasing densification and diversification of modern and upcoming wireless networks have become an important motivation for the development of agile spectrum sharing. Radio environment maps (REMs) are a basic tool for spectrum utilisation characterisation and adaptive resource allocation, but they need to be estimated through accurate interpolation methods. This work evaluated the performance of two established algorithms for spatial three-dimensional (3D) data collected in two real-world scenarios: indoors, through a mechanical measuring system, and outdoors, through an unmanned aerial vehicle (UAV) for measurement collection. The investigation was undertaken for the complete dataset on two-dimensional (2D) planes of different altitudes and for a subset of limited samples (representing the regions of interest or RoIs), which were combined together to describe the spatial 3D environment. A minimum error of −9.5 dB was achieved for a sampling ratio of 21%. The methods’ performance and the input data were analysed through the resulting Kriging error standard deviation (STD) and the STD of the distances between the measurement and the estimated points. Based on the results, several challenges for the interpolation performance and the analysis of the spatial RoIs are described. They facilitate the future development of 3D spectrum occupancy characterisation in indoor and UAV-based scenarios.

## 1. Introduction

**Figure 1:**
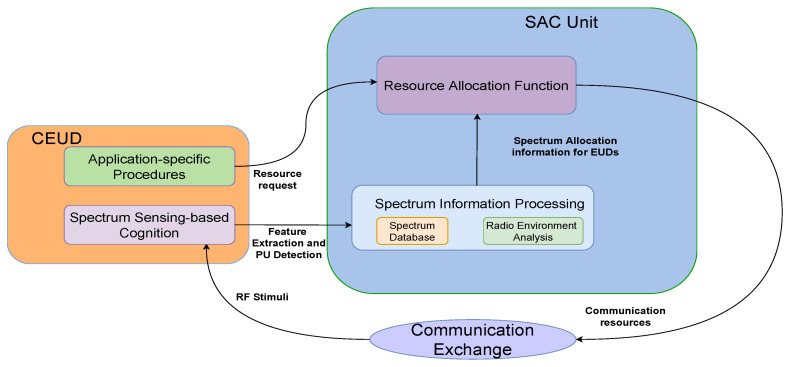
REMs within the scope of the HC^2^WA framework (simplified) [11].

The proliferation of wireless communications in more and more aspects of everyday life has led to increased advancements in bandwidth-intensive applications that require low latency and the agile processing of radio-frequency (RF) signals [1,2]. Naturally, addressing spectrum scarcity has gained more prominence in scientific research, in terms of multiple access methods, spectrum sharing, and spectrum utilisation characterisation for resource allocation that adapts to the radio environment as the users move within the area of a single plane (height) or in 3D space [3,4,5]. An important role in the development of such mechanisms is played by the application of a spectrum usage database through spatial REMs, as they allow for the computational relaxation of spectrum-sensing methods and the continual analysis of the spectral efficiency [6,7]. Due to the increasing deployment of dense communication nodes, such as Internet of Things (IoT) smart devices, femto-base stations (BSs), cellular users, and communication UAVs, the REMs can be utilised to aid the wireless technologies’ coexistence in a limited range of frequency bands, emergency communications, cellular offloading, spectrum decision and handover coverage optimisation, interference mitigation, and cognitive vehicle-to-everything (V2X) networks [6,8,9,10]. Thus, REMs represent an important functionality for the development of human-centric cognitive wireless access (HC^2^WA) toward the next-generation of communication networks [11]. The HC^2^WA is a conceptual framework of operation for spectrum-sensing-enabled cognitive radios (CRs) within the scope of the upcoming networks. The function of REMs within the HC^2^WA framework reflects the spectrum information exchange between the end user devices (EUDs) and the spectrum access control (SAC) units for the provision of services (Figure 1). The spectrum information collected by the EUDs is used to update the REMs, which comprise the spectrum database and enable the radio environment analysis. On the other hand, the available spectrum database allows the SAC unit to allocate the appropriate frequency and power resources for the communication exchange of the EUDs associated with it. This then drives the dynamics of the radio environment, which are perceived through the spectrum sensing of the EUDs.

Therefore, the dynamic change of spectrum utilisation in time, frequency, and 3D space should be considered, in order to characterise the resource availability. Respectively, the REMs need high accuracy even though they are to be estimated from a very limited number of samples, so as to achieve computationally light spectrum sharing [12]. This is necessary due to the substantial data volumes that the measurements would produce if the whole 3D region was covered by the sensor. On the other hand, some regions of the measurement volume are unreachable by the spectrum sensor, which makes the applicability of these solutions even more broad. Naturally, REM estimation from compressed sampling of the volumetric measurements has been a research topic with increasing importance in the field of terrestrial and airborne wireless communications [10,12,13,14,15,16]. A variety of both statistical-distance-dependence-based [10,12,13,14,15] and deep-learning-(DL)-based [16,17,18] interpolation methods have been developed for data collected in either 2D or 3D space that describes a large outdoor area. Additionally, for indoor scenarios, research in REM construction in 3D space has been limited, due to the difficulties in gathering measurements, which is feasible only through the use of specialised equipment [19]. Usually, in these scenarios, either movable objects or ray-tracing simulations are utilised to estimate the REMs in 2D space [20,21,22]. In spite of this substantive research work, the influence of the RF data (which themselves reflect the received signal’s dynamic propagation characteristics within an area at a certain height and within a volume of space) on the interpolation performance and the interpretability of the results has not been explored in great depth. This work considered real-world RF data from both an indoor scenario implemented via an automated mechanical system that allows movement in 3D space and an outdoor scenario implemented via UAVs. It expands on the authors’ previous research in [23], which evaluated several baseline interpolation algorithms for these two scenarios for the complete set of data in 3D space and described the methodology of their parameter calibration for optimal results. The evaluation in [23] established, empirically, that the two most-efficient baseline methods are Kriging and inverse distance weighting (IDW). In this work, they were evaluated for the cases of the complete set of measurements gathered in 2D space and for limited subsets, which constitute the RoIs, in 3D space. These regions comprise only the samples that reflect the significant variations in the received signal power. Therefore, they are utilised to estimate the rest. The ratio of the RoI samples relative to the overall number of measurements is defined as the sampling ratio [12].

The contributions of this work are as follows:

- An evaluation of interpolation efficiency was performed for RF data gathered in real-world indoor and outdoor scenarios, in 2D space for different altitudes, which provides insight into the exploration of the same methods in the case of spatial 3D data when only a limited number of samples is available. A normalised root-mean-squared error (NRMSE) of as little as −9.5 dB was achieved for a sampling ratio of 21%. The characteristics of the methods’ performance and the processed measurement samples were analysed by the Kriging error STD values and their densities and the STD of the distances between each measurement point and the estimated points.
- Several challenges for the future development of REM interpolation methods and for the analysis of spatial RoIs are described, on the basis of the signal propagation characteristics’ influence on the interpolation accuracy. These challenges facilitate the further investigation into the spatial 3D spectrum occupancy in indoor and UAV-based experimental setups.

The rest of this paper is organised as follows. A review of relevant literature sources is presented in Section 2. Then, Section 3 outlines the experimental setups for which the RF data were collected and the manner in which they were processed. Section 4 presents the evaluation results and their analysis, while the discussion on the challenges for future development is introduced in Section 5. Finally, the conclusion is established in Section 6.

## 2. Related Work

Research into methods for REM construction has expanded significantly to include measurement methodologies, interpolation in the temporal, spatial, or frequency domain, as well as application-specific topics, such as throughput maximisation, spectrum sharing, coverage mapping, and UAV path optimisation [6,24,25]. Most of the research in the field focuses on REM estimation based on 2D data (either real-world measurements or simulated) for outdoor scenarios [18,21,22,26,27,28,29]. The goal of these works is to either explore the methodology for the procurement of real-world measurements for REM construction and path loss estimation or develop a more-accurate interpolation method based on statistics/DL for the purpose of network coverage prediction and mapping in UAV–ground and UAV–UAV communications. In these works, REMs were constructed based on RF measurements from stationary cellular BSs and millimetre-wave (mmWave) sources. Additionally, REM reconstruction inferred on the basis of preliminarily generated REMs for a large variety of scenarios of transmitters and sensors’ placement was also suggested as a noteworthy approach, as it does not rely on particular path loss models [18]. Alternatively, a similar method was applied to infer the path loss from 2D REMs and floor plan images for different indoor environments using DL for REM estimation [20,21,22,29]. The literature has placed a particular focus on the spatial interpolation of REMs under a constrained number of samples, due to some influential works such as [10,12,13,14,15,18]. This section describes their main features and limitations, to outline the contributions of this paper. A summary of the reviewed publications that include interpolation methods, system models, applications, the achieved minimum error, and other details, is given in Table 1. These works focused primarily on enabling UAV-based communications and on measurement collection via flying nodes. This is due to the UAVs’ physical limitations (such as operational time and storage), which do not permit the procurement of the complete set of measurements that describe the radio environment in a certain 2D area/3D volume of space. The input data in these works were characterised by either limited measurements in 2D space (on a single plane) [16,17,18] or by combined sparse sets of measurements on a number of elevation levels (planes), with either the same or a random number of measurement samples per plane, in order to infer the radio environment in 3D space [10,12,13].

Alternatively, the input sparse measurements tensor can be vectorised to apply a modified compressed sampling method [15]. The proposed solutions utilised statistical (Kriging, tensor completion) and DL-based (autoencoders, generative adversarial networks) algorithms to estimate the REM on the basis of a small subset of the available data, described via the sampling ratio (usually below 25%) and are evaluated most often via the root-squared error (RSE) or RMSE metrics.

The specific goal of many of these works is to optimise the collection of measurements via UAVs, which also benefits from REM construction based on limited samples, as the aerial nodes have stringent operation time constraints and cannot fly into areas with large obstructions. Additionally, improving existing interpolation techniques for spectrum mapping in 3D space has also been explored for indoor locations based on measurements obtained via stationary SDR sensors [20,30].

This is common for the RF transmitter (TX) sources, the spectrum occupancy characterisation of which is described to be stationary and transmitting at a constant power level. At the same time, due to the difficulty and cost of the implementation of experimental setups that include UAV nodes, only data generated via deterministic models [10,12,14,15] or ray tracing [13,16,17,18] are usually employed. The relationship between the interpolation efficiency for the overall volume of space and the planes at different altitudes that comprise the volumetric data has not been sufficiently explored. In addition, the ascertainment of the RoIs in a deterministic manner also needs further investigation. It was, thus, the goal of this paper to evaluate the performance of some of the most-effective baseline estimation methods for limited training data and to analyse their dependence on the change of the measured signal’s power with altitude through the Kriging error STD and the STD between the estimated and the measurement samples. Both the scenarios of dense indoor stationary transmitters and that of outdoor multiple active UAVs were considered in the experimental data, which comprise the combined sparse sets of measurements on four planes with the same number of measurement samples per plane, for the inference of the radio environment in 3D space. For this purpose, real-world RF data collected in indoor and outdoor UAV-based communication scenarios for both fixed and airborne TX sources were utilised.

## 3. Experimental Setups and RF Data

### 3.1. Indoor 3D RF Measurements’ Scenario

**Figure 2:**
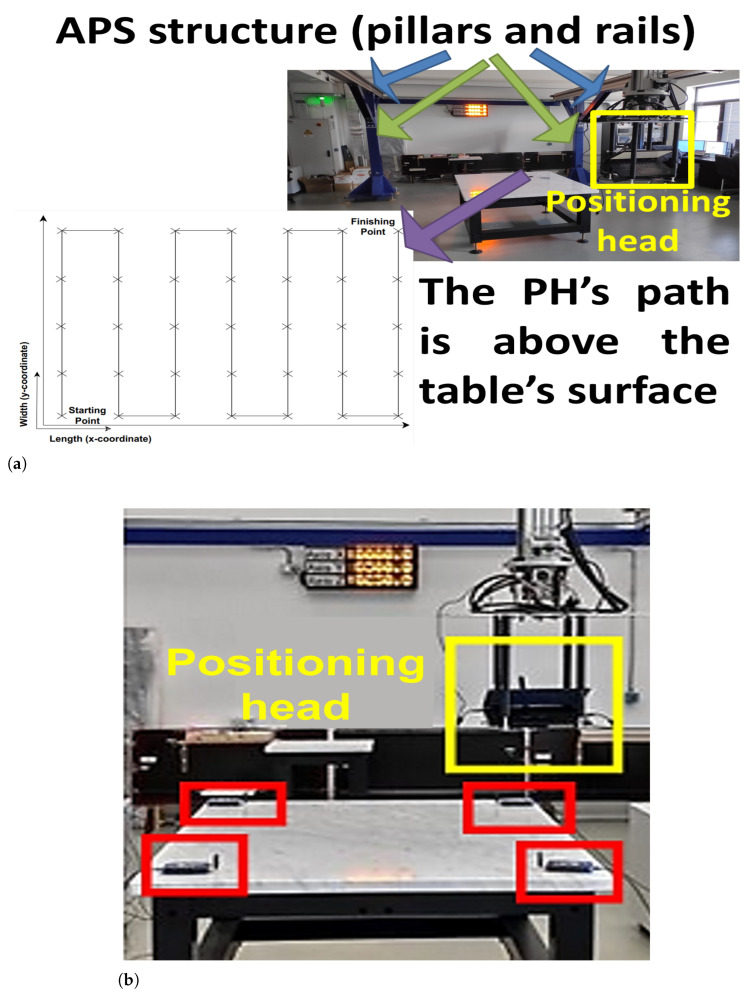
APS indoor experimental setup and scenario [24]. (**a**) APS structure comprising pillars (designated by the blue arrows) and rails (green arrows) that support the PH. The PH itself with the mounted SDR and host computer are in yellow; SDR transmitters are shown in red. The measurement path (designated by the purple arrow) is above the table, for a single level of elevation. (**b**) Experimental setup with SDR transmitters (in red); the PH is in yellow.

This experimental setup was realised in a large indoor location (Figure 2a) that included an automated positioning system (APS) and its robotic positioning head (PH) (shown in yellow), controlled by a remote computer, and the four SDRs, which transmit the source signal (shown in red in Figure 2b). Only the most-important details of the experimental setup will be described in this section, while a more-complete explanation may be found in the previous work [19].

The APS comprised four pillars that support the structure of the rails, which hold moving cylinders, which are elevated to 2.5 m above the ground (Figure 2a). They hold the PH, which is a mechanical unit, capable of mobility in the span of 10 m in the horizontal direction, 4 m in the vertical direction, and 1 m in height with a step of 0.1 m. In this experiment, the PH included a stand on which the host computer and the receiver SDR sensor were mounted. The Analog Devices (Norwood, MA, USA) PlutoSDR [32] was utilised. The host computer (Lenovo ThinkPad X250) that operated the SDR (via a USB 3.0 connection) had the following characteristics—a weight of 1.3 kg, a Core i3 5010U processor, 4 GB of random access memory (RAM), a solid-state drive (SSD) with a speed of up to 6 Gb/s, and operating on Ubuntu 20.04. The computer was manufactured by Lenovo (Bratislava, Slovakia). A separate computer, HP (Sofia, Bulgaria) Z230 Tower Workstation equipped with a Core i7 4790 processor, a hard disk drive with a speed of up to 6 Gb/s, and 4 GB of RAM, Windows 7, operated the APS to move the PH to the desired coordinates using a preliminary programmed route within a volume with dimensions of 6×2×0.75 m. The PH’s movement was performed in the following manner. Starting from the lowest height allowed by the APS, denoted as 0 m (30 cm above the surface of the table), the PH followed the path (shown in Figure 2a) along the length (*x*-coordinate) and width (*y*-coordinate), from the starting point to each consecutive one until the finishing location was reached, covering an area of 12 m^2^ (represented via $N_{P}$ = 35 points). There were no obstacles on the PH’s path and inside of the volume that was being considered in these measurements. At each location, the PH stayed for a duration of 7 s during which the SDR recorded the RF samples at the pre-defined SDR parameters (see below). Then, the PH would move to the next consecutive point. This procedure was repeated for each of the other three levels of elevation of 0.25, 0.5, and 0.75 m (above the lowest height). Thus, the whole volume, represented by the four sets of measurements, was covered. During the whole time span of this experiment, the four PlutoSDR transmitters, each placed at one corner of the table (shown in red rectangles in Figure 2b), emitted WiFi signals continuously. Each SDR was operated by a separate computer through GNU Radio (USB 2.0 connection). In addition, the receiver SDR was operated using a Python script, which, through GNU Radio, tuned the SDR and captured the received RF samples in a separate file for each measurement point.

The collected RF data were processed by dividing the measurement samples for each location into batches and taking the mean among the highest signal levels for each batch, as described in [19]. This mean value describes the received signal level at each location in space (i.e., the RF data). The RF parameters were as follows. A 5 MHz bandwidth at a central frequency of 2.484 GHz was covered, while omni-directional antennas with a gain of 4 dBi were utilised at both the transmitters and the receiver. The transmitters emitted orthogonal frequency division multiplexing (OFDM) WiFi signals with 64 subcarriers with binary phase-shift keying modulated symbols. The PlutoSDR software-defined radios (SDRs) were placed on the four corners of the table (as shown in Figure 2b), which delineated the volume covered by the APS. The transmission power was tuned empirically so that the instantaneous signal-to-noise ratio (SNR) of over 15 dB would be observed using the GNU Radio tools for the time and frequency analysis, while the SDR sensor was in a stationary position over the table’s centre. In other words, under this condition, the signals would be received clearly at the sensor. The average SNR of the received signal taken over the whole set of measurements was 24.15 dB. The experimental parameters are summarised in Table 2.

### 3.2. Outdoor 3D RF Measurements’ Scenario

**Figure 3:**
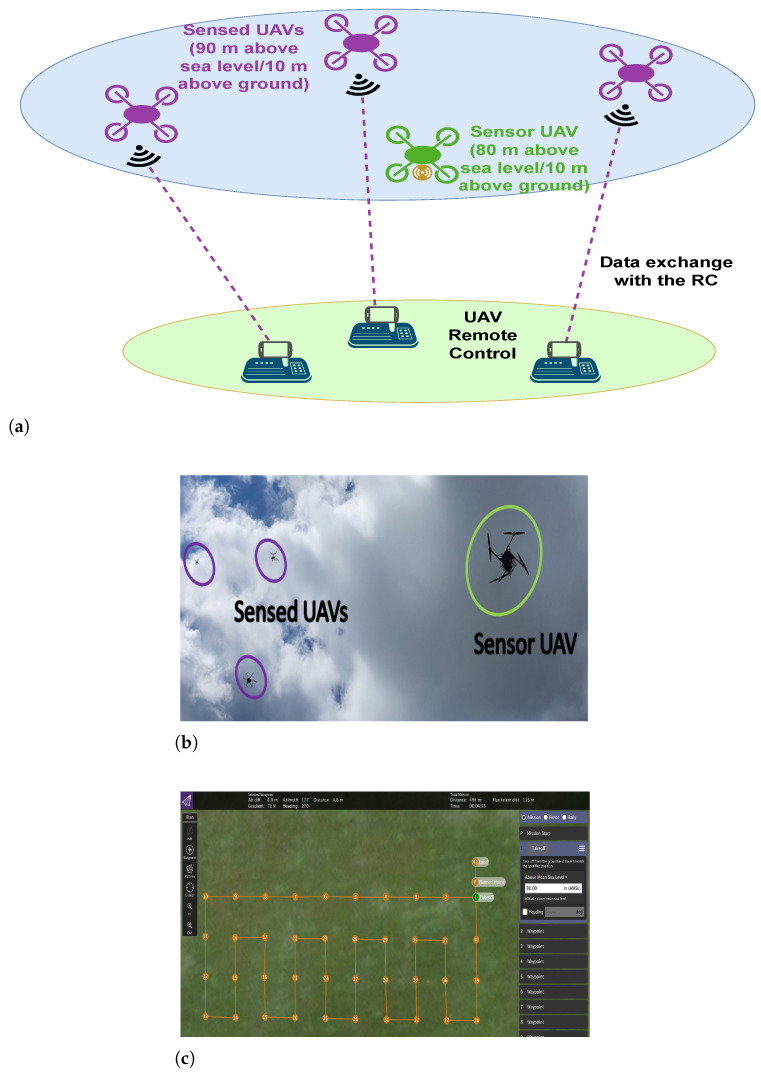
UAV-based experimental setup [24]. (**a**) General system model of the UAV scenario. The sensed UAVs are shown in purple, and their initial level is 90 m above sea level or 10 m above the sensor UAV (in green). (**b**) Sensor UAV (in green) and sensed UAVs (in violet) in flight. (**c**) Waypoint trajectory for the sensor UAV.

The experimental setup of the outdoor 3D UAV-based measurements’ scenario (Figure 3a,b) was implemented above the grounds of the Silkeborg El & Svæv field for model and UAV flights (near Silkeborg, Denmark). The UAVs employed for these experiments are the Freefly Systems (Woodinville, WA, USA) ALTA X and the DJI (Shenzhen, Guangdong, China) Matrice 600 Pro, Inspire 2, and Mavic 2 Enterprise Dual. The experiment comprised three “sensed UAVs” (DJI Matrice 600 Pro, Inspire 2, and Mavic 2 Enterprise Dual), which exchange control information with their ground-based remote controllers (RCs), and a separate “sensor UAV” (Freefly ALTA X), which measures the aforementioned signals through its on-board SDR (Nuand BladeRF 2.0 micro [33]). The sensed UAVs are termed as such because, as they performed their flight as a constellation, the signals which they emitted were being recorded for the 3D REM construction. The sensing was performed by the sensor UAV, which followed its own separate flight path, as described below. Again, only the basic details are given here, while the comprehensive description of the experiment is provided in the previous work [24].

The three sensed UAVs performed their flight in a constellation, playing the role of users of the spectrum. The sensor UAV followed a separate pre-determined path that comprised $N_{P}$ locations on four levels of altitude, each being 10 m lower than that of the constellation of sensed UAVs (illustrated in Figure 3a,b). The path it followed comprised 40 points, which constituted an area of 3990 m^2^ (105 m × 38 m (see Figure 3c)) at an (the lowest) altitude of 80 m above sea level (the ground was at an elevation of 70 m). The sensor UAV hovered at the coordinates of each point for 5 s before moving to the next one (the movement took around 4.7 s). The sensor UAV’s path was defined with the consideration of the area over which such flights were permissible and feasible, and based on this, the trajectories of the constellation of the sensed UAVs were defined. Before the start of the sensor UAV’s flight, a Python script would be executed, which would record the RF samples in a single file on the computer, using the GNU Radio tools for SDR tuning and reception. Afterwards, the sensed UAVs were set to start the execution of their trajectories together with the sensor UAV. This process was repeated for each of the three higher altitudes (90, 100, and 110 m above sea level). In the post-processing stage, the collected RF data at each altitude were processed so that the samples corresponding to each location along the sensor UAV’s measurement path may be determined. This was made possible through the sensor UAV’s logs, which recorded the timestamps of the beginning and end of the measurement path. Then, from these data, the mean over the samples that corresponded to the time period (5 s) was obtained, during which the sensor UAV hovered over each of the 40 positions, the coordinates of which were pre-determined, as shown in Figure 3c.

The following RCs were utilised for this experiment: Futaba T14SG (controls the Freefly ALTA X), DJI Matrice 600 Pro Radio Controller, DJI Inspire 2 Remote Controller, and DJI Mavic 2 Enterprise Dual Smart Controller. They operate at frequency bands in the ranges of 2.4–2.483 GHz and 5.725–5.825 GHz, which are used for telemetry, command and control, and video reception purposes for the UAVs. For waypoint trajectory planning and the execution of the sensor UAV, the missions were created using the QGroundControl software Version 4.2.0 and uploaded before launch. Figure 3c shows a snapshot of the waypoint trajectory planned in QGroundControl. It included the points of the takeoff, landing, and the 40 points that the sensor UAV covered during its flight.

To determine the frequency band that would be measured, the sensed UAVs were set into a preliminary flight trajectory (while the sensor UAV was offline), and through a manual search using the GNU Radio’s time and frequency analysis tools, a bandwidth that had significant and frequent emissions would be chosen. In this case, that was the 20 MHz bandwidth at a central frequency of 2.427 GHz. Mounted on the sensor UAV, the laptop Lenovo ThinkPad X250 (weight of 1.3 kg) operated the SDR via a universal serial bus (USB) 3.0 connection and was equipped with a Core i3 5010U processor, 4 GB of RAM, and a SSD with a speed of up to 6 Gb/s, under the Ubuntu 20.04 operating system. The Nuand (New York, NY, USA) BladeRF 2.0 micro SD is applicable for UAV-based measurements due to its compact dimensions (11 cm × 7.4 cm × 2.5 cm), weight of about 200 g, and its notable Analog Devices AD9361 transceiver frontend [34]. The SDR employed an omni-directional antenna with a gain of 2 dBi. The UAV RC signals had a proprietary structure based on OFDM WiFi (Lightbridge and OcuSync protocols) [35]. The average SNR of the received signal taken over the whole set of measurements was 18.81 dB. The experimental parameters are summarised in Table 3.

### 3.3. Experimental RF Datasets and Interpolation Methods

For both scenarios, the sensor SDR measured the spectrum at a central frequency $F_{C}$ and a bandwidth of *B* Hz (at a sampling frequency of $F_{S}$) for the whole period $T_{F}$, during which the measurement was performed, while *B*, $F_{C}$, and $F_{S}$ were considered as constants, i.e., interpolation was not performed in the frequency domain. As a consequence, $N_{S}=T_{F}F_{S}$ measurement samples were obtained, which define the bandwidth at every measured location within the space A that the experiment was performed in. Then, a subset $Z={\left\{z_{i}\right\}}_{i=1}^{N_{P}},Z\subset R^{3}$ of $N_{P}$ samples was selected, such that Z⊂A, and $z_{i}$ were the measurement points, which were used for the interpolation task. These samples comprised the received power $P\in R^{N}$ at each point in Z and formed the subset $S={\left\{P\left(z_{i}\right)\right\}}_{i=1}^{N_{P}}$. Each measurement location is described by the mean signal power $P(z_{i})$ for the measurement duration $T_{F}$ for a single point $z_{i},i\in Z$. The corresponding datasets of measurement points’ coordinates were of size $N_{x}\times N_{y}\times N_{H}$, where $N_{x}$ and $N_{y}$ denote the number of positions on the *x* and *y* axis, respectively, with $N_{x}=N_{y}$. The datasets of the measured signal levels and their coordinates were divided into *K* batches, which were used for the training and testing of the examined methods via the *K*-fold cross-validation technique. The points in each fold were selected across all elevation levels and shuffled randomly. As the evaluated methods required the relationships between the measurement points to be derived from the 3D distance *d* between them, the data points were converted into a size of $N_{P}^{\prime }\times 3$, where the $N_{P}^{\prime }$ distances were obtained as $d=\sqrt{{\left(x_{i+1}-x_{i}\right)}^{2}+{\left(y_{i+1}-y_{i}\right)}^{2}+{\left(z_{i+1}-z_{i}\right)}^{2}},\forall i\in Z$, with Z being the set of measurement points and *x*, *y*, and *z* representing the coordinates in the respective axes. The REM $\hat{R}$ can be defined as a function f(·), which was obtained from the power P measured at the points comprising the Z subset. This function usually involves the interpolation of the powers $\hat{P}$ at a set U∉Z of points, so as to increase the REM’s graphical resolution.

The interpolation itself was, in general, an estimation of P at an unknown point *u* using the measurement points $z_{i},i=\left\{1,\ldots ,N_{P}^{\prime }\right\}$, via an estimator described by the function F(·) in the following manner: $\hat{P}\left(u_{j}\right)=F\left[P\left(z_{i}\right),z_{i},u_{j}\right],\forall z_{i}\in Z,\;\forall u_{j}\in U$. The solution of this function involved the estimation of its parameters, denoted by $\eta \in R^{N_{P}},N_{P}\in N^{+}$. These parameters were obtained through a particular interpolation method, i.e., Kriging or IDW, using the methodology described in the previous work [23]. The theoretical derivations of these methods and their implementations were detailed in the literature [9,36,37]. Their basic outlines is briefly described below.

The IDW method considers the different influence of the known measured points on the estimated (unknown) ones, depending on the distance *d* between a predicted unknown point $u_{j}$ and all observed points $z_{i}$, as shown in (1):

(1) $$\hat{P}\left(u_{j}\right)=\frac{\sum_{i=1}^{N_{P}}{\left(d_{i}\right)}^{-k}P\left(z_{i}\right)}{\sum_{i=1}^{N_{P}}{\left(d_{i}\right)}^{-k}}\;,\;\forall z_{i}\in Z\;,\forall u_{j}\in U,$$

where *k* describes the weights’ decline with distance.

The Kriging method was implemented in the following steps:

- Choose a subset of $N_{P}$ points (out of the set Z) that are the closest to the unknown point *u*. These $N_{P}$ points are fit to an empirical variogram function f(h) (such as exponential, Matern, or spherical). The points’ values were obtained as a function of $P={\left.f(h)\right|}_{i=1}^{N_{P}}$ by dividing the $N_{P}$ points into subsets according to their distance from *u*.
- Then, for each subset, the variogram is described as in (2):
  (2) $$\gamma_{h}=\frac{1}{2n(h)}\sum_{i=1}^{n(h)}{\left(P\left(z_{i}\right)-P\left(z_{i}+h\right)\right)}^{2},$$
  where n(h) is the number of pairs of measured points that are separated by a distance *h*. Then, the variogram’s form, as well as its parameters (nugget, sill, and range) were determined empirically from the resulting function (as described in [23]).
- The Kriging weights η were computed by using the covariances between every two known points and between the estimated point and the observed points. These covariances were computed by equations that included the variogram’s form and depended on the distance *h* between every two points, the nugget, sill, and range [37]. Finally, the estimated power $\hat{P}$ was found by solving $\hat{P}\left(u_{j}\right)=P\eta$.

## 4. Simulation Results

For the two scenarios, the parameters related to the data and simulations are listed in Table 4. The data were processed in Python 3.9 (NumPy library, Jupyter Notebook environment) using a Lenovo ThinkPad X250 computer operating on Ubuntu, outfitted with a Core i3 5010U processor, 4 GB of RAM, and an SSD with a speed of up to 6 Gb/s.

The simulations for the complete set of measurements followed a similar methodology to the one described in [23] for the spatial 3D analysis of the collected data, which included the determination of the empirical variogram parameters (number of samples $N_{z}$, smoothness parameter ν, nugget, sill, and range) of the best variogram model. The evaluation was performed through Monte Carlo (MC) simulation of $N_{I}$ iterations; for each of these, a separate set of indices of the input measurement data, which comprises a subset of Z for each iteration, the percentage of training data $\Delta_{T}$ being fixed to 95%, was selected. This selection was performed for each individual plane of measurements. At each iteration, K-fold cross-validation was applied, i.e., 5% of all samples were selected at random, shuffled, and taken as points, the power of which would be estimated, while the rest were shuffled as well and used as known data for the estimation. This process was repeated until all folds of the samples had been considered. At the same time, two metrics were obtained: (1) the means of the resulting Kriging error STD $\sigma_{K}$, which measures the accuracy of the estimated $\hat{P}$ (i.e., it should be minimal [37,38]); (2) the STD $\sigma_{ME}$ of the distances between each measurement point and the estimated points. These two were averaged over all iterations at the end. The RMSE metric was normalised and defined as $NRMSE=\sqrt{\frac{\sum_{i=1}^{N_{U}}{\left(P_{i}-{\hat{P}}_{i}\right)}^{2}}{\sum_{i=1}^{N_{U}}{\left(P_{i}\right)}^{2}}}$, where $N_{U}$ is the number of points that were to be estimated. The normalisation was performed so as to represent the difference between the real value P of the point and its estimated value $\hat{P}$ regardless of the measured power’s order of magnitude. If the difference between the real and estimated value was small, the NRMSE was <0 (in dB), while if it was large (in comparison to the real value’s magnitude), the NRMSE would be >0 dB. The best variogram model was determined empirically from the 3D analysis in [23] to be the Matern type because it achieved the lowest $\sigma_{K}$ for both scenarios according to the methodology described in the same paper.

### 4.1. Evaluation of Spatial 2D Interpolation for the Complete Set of Measurements

**Figure 4:**
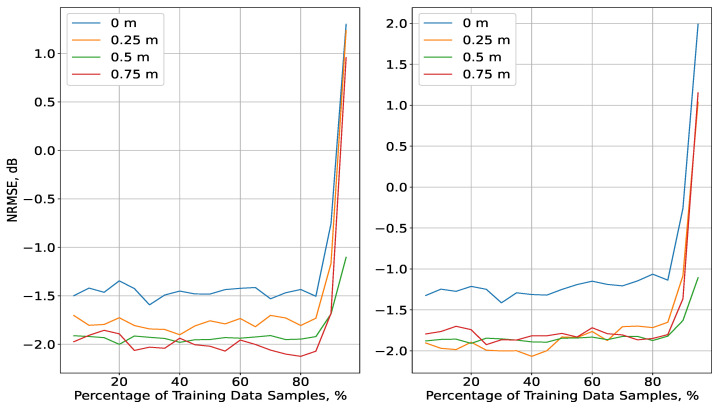
Evaluation of the IDW (**left**) and Kriging (**right**) interpolation methods depending on the percentage of training data $\Delta_{T}$ for the four planes in the indoor scenario.

Once the values of $N_{z}$, ν, and α that led to the smallest NRMSE for each plane were obtained, the overall values of these parameters were determined by taking their mean over all planes. They were as follows: $N_{z}=18$, ν=0.55, and α=3.17 for the indoor scenario and $N_{z}=15$, ν=1.3, and α=8.92 for the outdoor scenario. Then, the interpolation methods were calibrated, and they were evaluated for varying $\Delta_{T}$ between 5% and 95%, again in an MC simulation with K-fold cross-validation. The NRMSE for each plane was averaged over all folds for each iteration and, finally, over all iterations for a given $\Delta_{T}$. These simulations were performed only for the two most-accurate interpolation methods, found at the evaluation for the complete 3D data in [23], i.e., IDW and Kriging for both scenarios. The NRMSE results for the two interpolation methods (left for IDW, right for Kriging) for each of the four planes in the indoor scenario as a function of $\Delta_{T}$ are illustrated in Figure 4.

**Figure 5:**
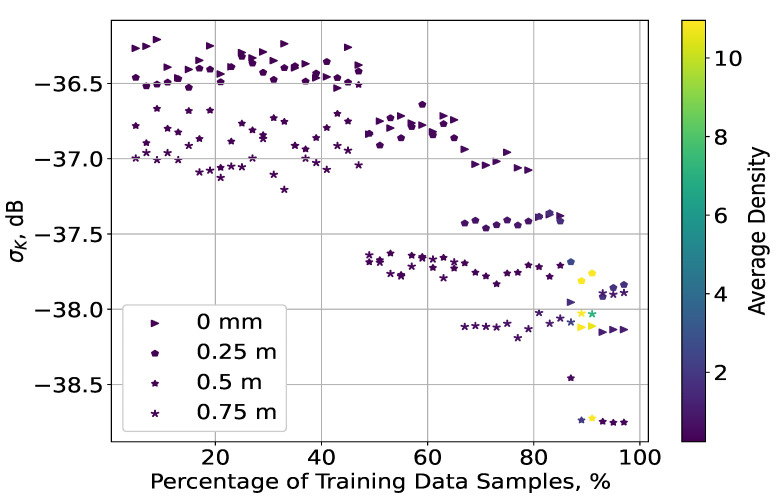
Distribution of the $\sigma_{K}$ values and their densities as functions of $\Delta_{T}$ (indoor scenario).

The results for both methods had very similar performance, and their NRMSE curves followed the same distributions. It is notable that the accuracy was about 0.5 dB smaller for the measurement data gathered at the lowest level of height and that the IDW method yielded about a 0.2 dB improvement, compared to Kriging. In addition, for the IDW case, there was a slight increase in the error at a height of 0.25 m, while for the two highest levels, there was very little variety, which also held for the results at 0.25, 0.5, and 0.75 m for the Kriging method. The NRMSE had negligible variations for $\Delta_{T}\in \left[5;90\right]\%$, while, notably, there was a considerable degradation of accuracy by about 3.5 dB for $\Delta_{T}>90\%$. To explore the reason for this development, the distribution of the Kriging error STD $\sigma_{K}$ as a function of $\Delta_{T}$ was explored. Thus, the densities of $\sigma_{K}$ (and its corresponding values) of all *K* folds were computed, which were then averaged for each plane over all histogram bins at each iteration. Finally, the mean of the STD values and their densities over all iterations were obtained, and their relationship is illustrated in Figure 5, with the change in density represented by the range of colours (as shown in the colour map). The STD values for the four planes declined close to exponentially with $\Delta_{T}$, and they were distributed very similarly. The density of low STD values for $\Delta_{T}$ around 90% was much higher than for the rest of the range, which led to a significant change in the NRMSE results. However, due to the lack of consistency in this tendency (i.e., the density declined drastically) as $\Delta_{T}$ increased, the error continued to grow, as is visible in Figure 4. In other words, the amount of low $\sigma_{K}$ values for $\Delta_{T}>90\%$ was comparable to that of the much higher $\sigma_{K}$ values that were achieved for $\Delta_{T}\in \left[5;85\right]\%$. Despite the close to exponential decline of $\sigma_{K}$ with $\Delta_{T}$, the exceptional change in its density, which was not retained for $\Delta_{T}>90\%$, led to an increasing NRMSE.

**Figure 6:**
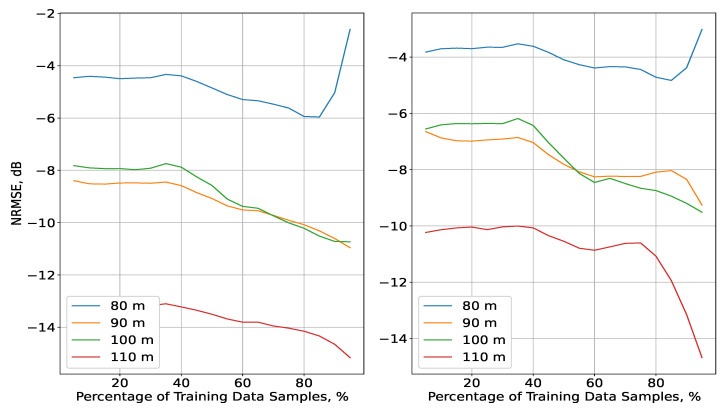
Evaluation of the IDW (**left**) and Kriging (**right**) interpolation methods depending on the percentage of training data $\Delta_{T}$ for the four planes in the outdoor scenario.

**Figure 7:**
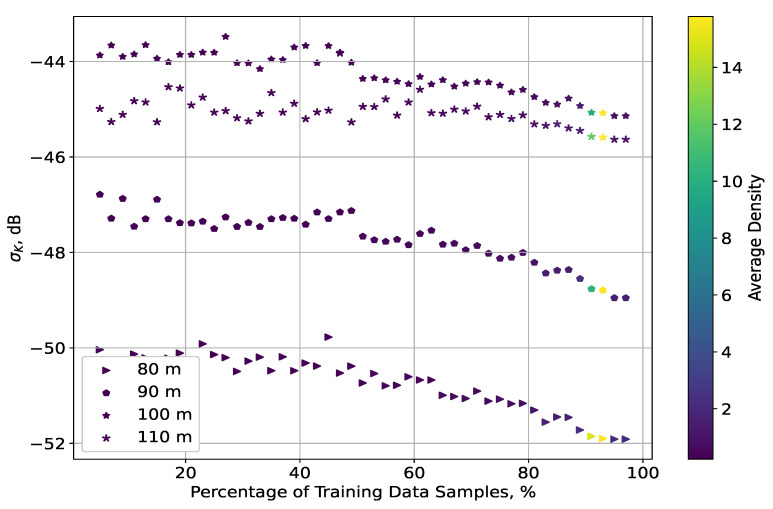
Distribution of the $\sigma_{K}$ values and their densities, as a function of $\Delta_{T}$ (outdoor scenario).

As for the outdoor scenario, the results are presented in Figure 6. Overall, the accuracy increased with height, following the conclusions drawn from [24]. Just as in the alternative scenario, the results for both methods were similar to one another in terms of their distributions, with the IDW interpolation achieving up to a 2.5 dB gain for $\Delta_{T}<90\%$. A notable difference in the NRMSE distributions in comparison to the indoor scenario was observed for all planes apart from the one with the lowest altitude (80 m). The error was highest, with a slight decline for $\Delta_{T}\in \left[5;80\right)$, followed by a growth for the highest values of $\Delta_{T}$. In contrast, the NRMSE for the planes of altitudes above 80 m exhibited consistent declines, which were most significant for $\Delta_{T}>80\%$, with the results for altitudes of 90 and 100 m being very close to each other. In addition, the interpolation at the highest plane also had the best accuracy. To explore these developments further, an analysis based on the distribution of $\sigma_{K}$ (Figure 7) was performed in the same manner as for the indoor scenario.

Distinct from the error STD in the indoor scenario, the same metric here differed significantly with the change of altitude. For each plane, $\sigma_{K}$ declined with the increase of $\Delta_{T}$ and was separated from the results for the other planes. Nevertheless, the STD distributions for altitudes above 80 m were much closer to each other, differing from each other by less than 2.5 dB. In addition, the density of their STD values changed insignificantly between themselves: this was characterised as nearly constant for the whole range of $\Delta_{T}$, apart from the interval $\left(90;95\right)\%$, in which there were two consecutive increments in the density.

This uniformity for the planes at altitudes of 90, 100, and 110 m led to the similar distributions of their NRMSEs. A drastic growth for $\Delta_{T}\in \left[90;100\right)\%$ was not present because the corresponding upsurges in the $\sigma_{K}$ density were not as substantial as those for the plane at 80 m. This was particularly due to the intensity of these increases in density, such that the NRMSE grew for the highest $\Delta_{T}$ in a similar manner to the results for the indoor scenario. The values of $\sigma_{K}$, themselves being the lowest for this plane, did not affect the accuracy as much as the variations in the received signal power, which were much more significant at the lowest altitudes [24].

### 4.2. Evaluation of Spatial 3D Interpolation for a Limited Set of Measurements

In this subsection, on the basis of the preceding experiments performed both in [23] and above, the IDW and Kriging interpolation methods were evaluated for limited measurements in terms of the RoIs, which were separately defined for each plane and combined to perform the estimation in 3D space. The RoIs were determined by the absolute differences of every two samples for each plane, and those that differed by more than 0.5 dB defined the RoI for that plane. They were, then, combined in a single batch of measurement points, and an MC simulation was performed, which included the whole batch as the input and iterated over $N_{u}$ unknown points for all planes (which were also combined), starting from one to the overall $N_{U}$. At each iteration, the indices of the measurement and of the estimated points were shuffled. For the indoor scenario, the whole range of $N_{P}$ samples for each plane differed by over 0.5 dB and varied significantly, which made the identification of the RoIs impossible using this approach. Nevertheless, due to the nearly constant NRMSEs for both the 2D (as observed in Section 4.1) and 3D (in [23]) data for $\Delta_{T}=\left[5;40\right]\%$ in the indoor scenario, it makes sense to take a small number of samples and evaluate the interpolation accuracy for the rest of the points using the methodology described above. Thus, 20% of all $N_{P}$ points for each plane were randomly selected and combined, and the interpolation was performed for the rest. As for the outdoor scenario, as described in [24], the received signal variations were very significant in the lowest planes (80 and 90 m), and their intensity declined with altitude. Using the RoI determination method described above, it was found that, for the planes at altitudes of 80, 90, and 100 m, the RoI samples were, respectively, 63, 12, and 5% of the overall $N_{P}$ points for each plane. Due to the comparatively very little variations for the measurements at 110 m, the RoI was determined by choosing 5% of the samples at random.

**Figure 8:**
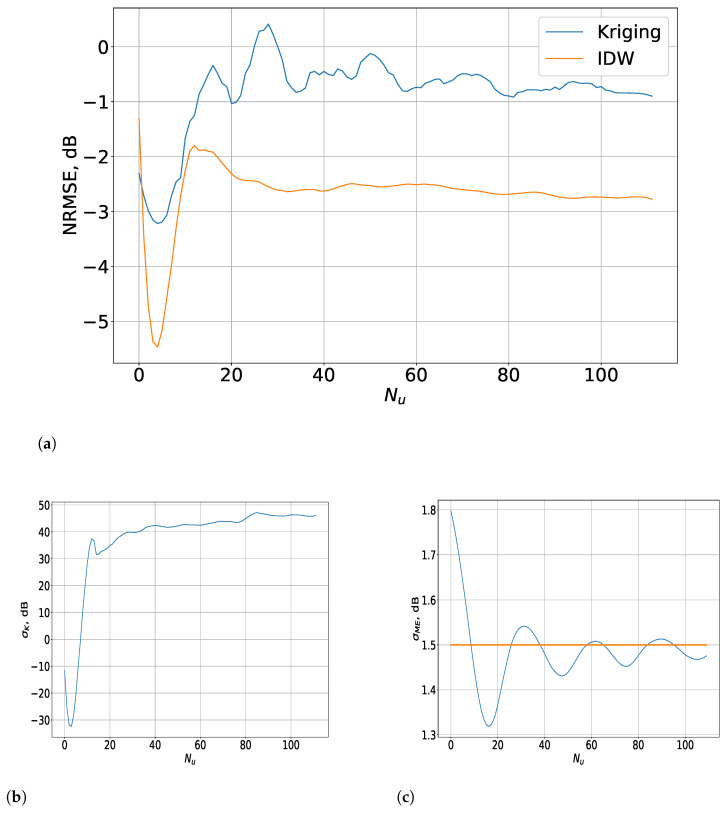
Results for the interpolated 3D measurements from a limited set of samples for the indoor scenario. (**a**) Evaluation of the IDW and Kriging interpolation methods depending on $N_{u}$ for spatial 3D data. (**b**) Distribution of the $\sigma_{K}$ values depending on $N_{u}$. (**c**) Distribution of the $\sigma_{ME}$ values depending on $N_{u}$.

**Figure 9:**
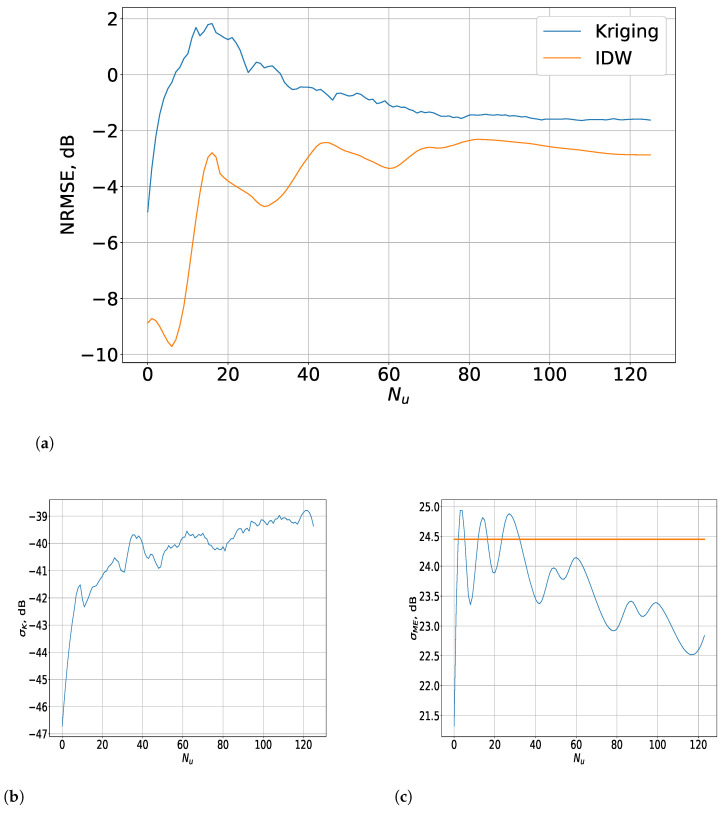
Results for the interpolated 3D measurements from a limited set of samples for the outdoor scenario. (**a**) Evaluation of the IDW and Kriging interpolation methods depending on $N_{u}$ for spatial 3D data. Evaluation of the IDW and Kriging interpolation methods depending on $N_{u}$ for spatial 3D data. (**b**) Distribution of the $\sigma_{K}$ values depending on $N_{u}$. (**c**) Distribution of the $\sigma_{ME}$ values depending on $N_{u}$.

The results for the interpolated 3D measurements from a limited set of samples are presented in Figure 8 (for the indoor scenario) and Figure 9 (for the outdoor one).

As described above, the NRMSE was evaluated for a varying number of estimated samples $N_{u}$, while the limited sets of measurement points that describe the RoIs remained the same. As observed for the results with the input of the complete 3D data in [23], for a small $\Delta_{T}$, the IDW method outperformed Kriging for a limited set of RF data, due to its small weight decline coefficient. Thus, the power of the more-distant points was also considered as non-negligible, which improved the IDW interpolation accuracy in the case of a small inter-point distance, natural in this scenario. There was a drastic decline in the NRMSE with about 4 dB for $N_{u}<10$, which was followed by a fast convergence at around −2.8 dB at $N_{u}=70$. On the other hand, the accuracy for the Kriging method was about 1.5 dB lower and followed a similar distribution with the distinction that there was much greater variation (within a span of 1.2 dB) for $N_{u}\in \left[10;80\right]$ before the function reached relative stability.

To analyse these relationships, the STD $\sigma_{ME}$ of the distances between each measurement point and the estimated points and the average STD $\sigma_{K}$ of the estimated Kriging error were considered. The former showed a declining function followed by alternating peaks of small variation, which is relevant to the fluctuations of the NRMSE for $N_{u}<20$ because the overall mean STD $\bar{\sigma }$ (shown as a straight line) of the complete set of $N_{P}^{\prime }$ points was 1.5 m. Thus, it follows that the initial decrease in $\sigma_{ME}$ to 10% below $\bar{\sigma }$ was related to the drastic decline in the NRMSE for the IDW method. Further, the function started to vary around the mean value, thus establishing the increase in the NRMSE until convergence was reached. In addition, $\sigma_{K}$ showed a prominent decline, followed by a drastic increase for $N_{u}\in \left[10;20\right]$, which retained a very high STD in the region of $N_{u}\in \left(20;80\right]$, after which it increased slightly and converged. This substantial increase in $\sigma_{K}$ compared to the same metric for $N_{u}\in \left(0;10\right)$ led to a high NRMSE with notable fluctuations, which became negligible for $N_{u}>80$.

In the outdoor scenario, the NRMSE exhibited some distinct characteristics in comparison to the alternative, even though the IDW was more accurate than Kriging. The difference between them was nevertheless much smaller, with it being substantial only for $N_{u}<15$. In that region, just as in the case of the Kriging method, the IDW’s accuracy declined significantly with the increase of $N_{u}$. Contrasted with the alternative, the IDW showed noticeable variations in not only this interval, but also for $N_{u}\in \left[15;60\right]$. The reason for this can be determined from the results for $\sigma_{ME}$ (Figure 9c), which illustrate that, for most of the interval of $N_{u}\in \left[1;15\right)$, $\sigma_{ME}$ was much smaller than $\bar{\sigma }$ (shown as a straight line), which was 24.5 m in this case. Nevertheless, $\sigma_{ME}$ increased and varied around the overall mean, which led to the fluctuations of the NRMSE for $N_{u}\in \left[15;40\right]$. They became less intensive until convergence was reached at an NRMSE of −2.8 dB for $N_{u}\geq 70$ as the $\sigma_{ME}$ declined, although the error’s variations still retained some of their intensity.

On the other hand, the NRMSE of the Kriging method rose significantly for $N_{u}<15$, after which it declined steadily until convergence was reached for $N_{u}\geq 70$. At that point, the difference between the Kriging and IDW error was about 1 dB. The results for $\sigma_{K}$ differed substantially from those for the indoor scenario in terms of its order of magnitude, which changed only within the span of several dB. Its increase was steady for $N_{u}<15$, after which the variations became much less intensive and the function tended to convergence for $N_{u}\geq 70$. The small change of $\sigma_{K}$ led to the steady decline in the NRMSE as $N_{u}$ increased.

As observed from the results of both scenarios, the fluctuations of $\sigma_{ME}$ affected the NRMSE not only of the IDW but of Kriging as well. Its relationship to the overall mean STD $\bar{\sigma }$ determined the sharp declines of the accuracy, while the intensity of its variations influenced the number of estimated samples for which the NRMSE converged. This value of $N_{u}$ was the threshold for the interpolation method’s efficiency as the accuracy was retained regardless of the increase in this parameter, while the amount of measurement samples was still relatively small.

## 5. Discussion

The experimental results presented in Section 4 show the following aspects of the interpolation performance and the RF data analysis for REM construction:

- Both the Kriging and IDW methods converged to an NRMSE below 0 dB, which makes them suitable for limited real-world 3D RF data, and the achieved accuracy was not significantly lower than in the case where the complete set of measurements was available (shown in the previous work [23]). In comparison, the NRMSE was roughly the same for the indoor scenario, or about 4 dB higher for the outdoor scenario. As observed in Figure 8 and Figure 9, more-consistent accuracy was attained in the outdoor scenario where the RoIs can be more appropriately defined. That was due to the clear distinction between the clusters of measurements with small variation (below 0.5 dB) between every two consecutive points and those that differed noticeably (i.e., which constituted the RoIs). Stable convergence was achieved at an NRMSE of −2.8 dB for $N_{u}\geq 70$ in both scenarios. As indicated by the significant change in the STD, the Kriging method yielded much-less-stable results if a random subset of points (in the indoor scenario) was used for the interpolation. In general, the STD of the distance between the measured and the estimated points had a notable influence on the performance of both methods.
  ***Relevant challenge:*** To increase the interpolation efficiency for REM construction, precise mechanisms for RoI determination should be developed, such that the characteristics of the environment are considered. The two scenarios for which the interpolation was evaluated were characterised either by fast-changing (difference of below 0.5 dB between every two measurements) received signal power throughout the entire measurement volume (indoor scenario) or by the notably slower rate of change for most of the measurements (outdoor scenario). The RF data for the former scenario were influenced by the experimental setup of a slow-moving receiver in an indoor environment (within a volume of 6 × 2 × 0.75 m), which included severe multipath propagation, thus requiring an even higher resolution of the measurements [23]. On the other hand, the outdoor scenario comprised a much-more-spacious area (105 × 38 m) at each height, and thus, the distance between each two points, although constant, was significantly greater than for the alternative. The points were adequately dispersed in space and sufficient in number, such that, for most values of $N_{u}$, $\sigma_{ME}$ was below the average, thus emphasising the importance of the appropriate selection of (even a small subset of) samples for the RoI, while the rest can be reliably estimated. This can be achieved by adaptive mechanisms for upsampling-based interpolation [12], sensing location estimation through orthogonal matching pursuit [15], autoencoder-based tensor completion [16,39], and statistical approximation of the signal propagation [10] or of the LoS availability [40].
- As observed from the previous works [19,24], which introduced the experiments, the intensity of the received signal variations decreased with height, even though this phenomenon had much lesser influence in the indoor scenario, as the rate of change remained high for all planes. Following this, the NRMSE distributions (Figure 4 and Figure 6) for both interpolations reflected these observations as the results for different planes in the indoor scenario were very close to each other, while their accuracy was much lower. In contrast, the error for the four planes in the outdoor scenario was distributed in such a manner that there was a notable difference between the lowest and highest plane and the two planes between them. Figure 4 and Figure 6 show that the decline in the NRMSE was not necessarily consistent with the increase in height. For the indoor scenario, the NRMSE at 0.75 m was very close to those at 0.25 and 0.5 m. In contrast, the NRMSE in the outdoor scenario was 3–5 dB lower at the highest altitude (110 m) in comparison to the measurements at the planes of 90 and 100 m, which had a negligible difference from each other. The signal power fluctuations, therefore, affected the accuracy and the stability of its distributions for each plane, such that the lower the variations, the more consistent the NRMSE would remain with the increase of the percentage of training data $\Delta_{T}$.
  ***Relevant challenge:*** The environment in which the spatial 3D measurements are collected needs to be considered in the planning of the experimental setup and in the design of the interpolation and prediction methods. Measurement planes at low heights require more measurement samples (i.e., shorter distance between every two measurements), especially in an indoor environment. Therefore, an algorithm for multipath propagation prediction can be employed to identify the RoIs and optimise the measurement collection at multiple heights, while its results are validated through preliminary measurements on a single plane. The novel generative adversarial networks and convolutional and graph neural networks [20,21,41,42,43] are some appropriate solutions for that purpose, thus reducing the number of measurement points and increasing the accuracy. An advantage of these methods is that they can utilise existing simulated or real-world signal measurement datasets to predict the RoIs [18]. Such methods have mostly been used for 2D RF data on a single plane and will need to be adapted to account for the signal variations in 3D space [16,24].
- The adequacy of available RF datasets for REM construction should be evaluated depending on the scenario for which the interpolation is to be performed. There are several types of datasets that have been commonly utilised in recent literature—mobile networks in various outdoor areas [16,43], UAV-based sensing and coverage provision [24,40], and simulated and real-world indoor data for stationary receivers [18,21,39]. To the best of the authors’ knowledge, the efficiency of these datasets for REM estimation in various environments and scenarios, different than the ones within the scope of their individual papers, has not been established.
  ***Relevant challenge:*** In order for more-accurate and -adaptable interpolation methods to be developed, there is a need for: (1) the generation of sufficiently large datasets for each relevant scenario; and (2) the study of the current datasets’ adequacy for training in more challenging scenarios with multiple transmitter and receiver types. Both of these aspects are relevant to the field of spectrum occupancy characterisation through REM and spectrum sensing, but each has its own challenges. The generation of datasets in complex scenarios may often be very difficult and/or expensive, especially if the experimental setup includes multiple UAVs. On the other hand, the usage of publicly available datasets necessitates the further analysis of their features (such as how the signal varies in time or space), which is to be considered in the design of REM prediction and interpolation algorithms, and in the interpretability of their results, which is of great interest for resource allocation and network planning.

## 6. Conclusions

This paper presented an evaluation of two of the most-prominent interpolation methods (Kriging and IDW) for RF data collected in two relevant real-world scenarios. The complete sets of measurements in 2D space and for the limited subset in 3D space were considered to provide further insight into the estimation performance for REM construction. The in-depth analysis in 2D space facilitated the determination of the RoIs for each dataset, which were afterwards used for the latter simulation. The variations in the interpolation performance in height and in terms of $N_{u}$ were described using the STD of the distances between the measurement and the estimated points, as well as the Kriging error STD. Based on these results, several aspects of the RF data analysis and REM interpolation accuracy were characterised, and important directions for future work were outlined. In summary, it was established that inconsistencies in the distribution of $\sigma_{ME}$, which were significant in comparison to the overall data mean STD $\bar{\sigma }$, led to an uncharacteristic growth in the NRMSE, regardless of the interpolation method’s type. Additionally, the consideration of the RoIs for interpolation using limited samples is adequate, but its efficiency depends on the specific scenario. The results give important insights into the challenges for the future exploration of the spatial 3D spectrum occupancy in indoor and outdoor UAV-based experimental setups and of the interpolation methods used for that purpose.

## Figures and Tables

**Table 1:** Summary of recent works in the field of interpolation for REM construction.

Reference, Year	Interpolation Method and Research Topic	Scenario	Interpolation Performance	Notes
[10], 2022	Statistical geometric modelling for UAV path optimisation	Simulated 3D data; multiple stationary TX sources	Relative error of −10 dB at a sampling ratio of 14%	Wireless coverage mapping
[12], 2020	IDW method; UAV path optimisation for the IoT	Simulated 3D data; multiple stationary TX sources	RSE of −8 dB at a sampling ratio of 25%	RoI-based optimisation
[13], 2021	Model-driven statistical signal inference from limited samples; UAV path optimisation	Simulated 3D data; single stationary TX source	Normalised RSE of −11 dB at a sampling ratio of 20%	-
[14], 2022	Alternating direction method of multipliers method; UAV path optimisation	Simulated 3D data; multiple stationary TX sources	RSE of −5 dB at a sampling ratio of 30%	-
[15], 2021	Orthogonal matching pursuit optimisation; UAV path optimisation for IoT	Simulated 3D data; multiple stationary TX sources	RSE of −8 dB at a sampling ratio of 20%	Statistical modelling
[16], 2021	Deep autoencoder for tensor completion; UAV path optimisation	Simulated 2D data; multiple stationary TX sources	RMSE of 5 dB at a sampling ratio of 10%	-
[17], 2022	Deep autoencoder combined with a Bayesian estimator; UAV path optimisation	Simulated 2D data; multiple stationary TX sources	RMSE of 4 dB at a sampling ratio of 6%	Data-distribution-driven algorithm design
[18], 2023	Deep-CNN-based GAN for cellular coverage estimation in multiple environments	Simulated 2D data; multiple stationary TX sources	RMSE of 6 dB at a sampling ratio of 5%	Analysis of different distributions from which the test data were sampled
[21], 2022	DL-based interpolation	Simulated 2D data; single stationary WiFi source	RMSE of 11.8 dB	Graph-neural-network-based method
[27], 2021	Path loss and antenna pattern estimation; mmWave measurement system	Real-world 2D data; single stationary TX source	Not applicable	Custom-made UAV with RF measurement capability
[28], 2021	UAV flight optimisation and coverage prediction	Simulated 2D data; Multiple stationary cellular BSs	MSE of −20 dB	Reinforcement learning
[30], 2019	Kriging method; TV broadcast coverage mapping	Real-world 3D data; single stationary TX source	RMSE of 4.5 dB	-
[31], 2022	Cellular coverage mapping and network performance assessment	Real-world 3D data; single stationary 5G BS	Not applicable	Custom-made UAV with RF measurement capability
This work	Kriging and IDW; indoor and outdoor measurements for spectrum occupancy characterisation	Real-world 3D data for outdoor UAV and indoor scenarios; multiple stationary and flying UAV TX sources	NRMSE of −9.5 dB at a sampling ratio of 21%	Analysis of the STD in 2D and 3D for data-driven interpolation

**Table 2:** Experimental and RF parameters for the APS scenario.

Parameter	Values
Dimensions of experimental volume, m	6×2×0.75
*B*, MHz	5
$F_{C}$, MHz	2484
$F_{S}$, MHz	10
$N_{P}$	35
Number of emitters/signal type	4/WiFi
Transmission power, dBm	−3

**Table 3:** Experimental and RF parameters for the UAV scenario.

Parameter	Values
Dimensions of experimental volume, m	105×38×30
*B*, MHz	20
$F_{C}$, MHz	2427
$F_{S}$, MHz	40
$N_{P}$	40
Number of emitters/signal type	3/proprietary variants of WiFi
Transmission power, dBm	≤20 (Matrice 600 Pro), ≤26 (Inspire 2 and Mavic 2 Enterprise Dual)

**Table 4:** RF data and simulation parameters for the two scenarios.

Parameter	Values		Parameter	Value Range
	*Indoor (APS) *	*Outdoor (UAV)*		
*B*, MHz	5	20	$\Delta_{T}$, %	$\left[5;5;95\right]$
$F_{C}$, MHz	2484	2427	$N_{z}$	$\left[5;5;100\right]$
$N_{P}$	35	40	ν	$\left[0.05;0.5;9\right]$
$N_{P}^{\prime }$	140	160	α	$\left[0.05;0.5;9\right]$
$N_{H}$	4	4	$N_{I}$	100

## Data Availability

The data for the replication of the results are available from the authors upon request.

## Abbreviations

The following abbreviations are used in this manuscript:

2D	Two-dimensional
3D	Three-dimensional
APS	Automated positioning system
CR	Cognitive radio
DL	Deep learning
EUD	End user device
HC^2^WA	Human-centric cognitive wireless access
IDW	Inverse distance weighting
IoT	Internet of Things
MC	Monte Carlo
NRMSE	Normalised root-mean-squared error
OFDM	Orthogonal frequency division multiplexing
PH	Positioning head
RAM	Random access memory
RC	Remote controller
REM	Radio environment map
RF	Radio frequency
RSE	Root-squared error
SAC	Spectrum access control
SDR	Software-defined radio
SNR	Signal-to-noise ratio
SSD	Solid-state drive
STD	Standard deviation
TX	Transmitter
UAV	Unmanned aerial vehicle
V2X	Vehicle-to-everything
